# Supplementary material for: Cohesion is established during DNA replication utilising chromosome associated cohesin rings as well as those loaded de novo onto nascent DNAs
Source: eLife. 2020 Jun 9;9:e56611. doi: 10.7554/eLife.56611 (PMC7282809; doi:10.7554/eLife.56611)
Supplement: Supplementary file 1. [file elife-56611-supp1.docx]

| Genotype | Source | Strain No. |
| --- | --- | --- |
| *S. cerevisiae MATa ade2-1 trp1-1 can1-100 leu2-3,112 his3-11,15 ura3 GAL psi+*  *All following strains are based on this background* | This study | **K699** |
| *S. cerevisiae*  *SK1 MAT a/alpha*  homozygous for:  *ho::LYS2 promURA3::tetR::GFP-LEU2 REC8-HA3::URA3 ADE2*  *his3::hisG trp1::hisG PDS1-myc18::TRP1(K.lactis)*  heterozygous for:  *tetOx224-URA3* | This study | **K10003** |
| *S. cerevisiae*  *SK1 MAT a/alpha*  homozygous for:  *ho::LYS2 ctf8::KAN promURA3::tetR::GFP-LEU2 REC8-HA3::URA3 PDS1-myc18::TRP1(K.lactis),*  *ADE2 his3::hisG trp1::hisG*  heterozygous for:  *tetOx224-URA3* | This study | **K10349** |
| *S. cerevisiae*  *SK1 MATa/alpha*  homozygous for:  *ho::LYS2 promURA3::tetR::GFP-LEU2 REC8-HA3::URA3*  *ADE2 his3::hisG trp1::hisG*  *PDS1-myc18::TRP1(K.lactis)*  *ctf4::KanMX*  heterozygous for:  *tetOx224-URA3* | This study | **K11692** |
| *S. cerevisiae MATa Smc1(G22C,K639C)::NatMX4 Smc3(E570C,S1043C)::ADE2 leu2::Gal-Scc1(R180E,R268D, A547C)-PK6::LEU2 SCC3 (K404E)-HA3::HIS 2.3 kb Trp1-ARS1-Cen4 plasmid* | This study | **K24697** |
| *S. cerevisiae MATa Smc1(G22C,K639C)::NatMX4 Smc3(E570C,S1043C)::ADE2 leu2::Gal-Scc1(R180E,R268D, A547C)-PK6::LEU2 scc3 (K404E)-HA3::HIS scc2-45::natMX (L545P D575G) TRP1-ARS1-CEN4 2.3KB plasmid* | This study | **K24738** |
| *S. cerevisiae MAT a*  *Smc3 (E570C,S1043C)::ADE2*  *Smc1(G22C,K639C)::natMX*  *leu2::Gal-Scc1(R180E,R268D A547C)-PK6::LEU2*  *chl1::KanMX*  *scc2-45:NAT*  *scc3K404E::His3MX 2.3kb Trp1-ARS1-Cen4 plasmid* | This study | **K28061** |
| *S. cerevisiae*  *MAT alpha*  *Scc1(A547C)-pk6::Kan*  *Smc3 (E570C,S1043C)::ADE2*  *Smc1(G22C,K639C)::NAT*  *chl1::His3MX 2.3kb Trp1-ARS1-Cen4 plasmid* | This study | **K28082** |
| *S. cerevisiae*  *MAT alpha*  *Scc1(A547C)-pk6::Kan*  *Smc3 (E570C,S1043C)::ADE2*  *Smc1(G22c,K639C)::NAT*  *ctf4::His3MX 2.3kb Trp1-ARS1-Cen4 plasmid* | This study | **K28084** |
| *S. cerevisiae*  *MAT alpha*  *Scc1(A547C)-pk6::Kan*  *Smc3 (E570C,S1043C)::ADE2*  *Smc1(G22c,K639C)::NAT*  *tof1::His3MX 2.3kb Trp1-ARS1-Cen4 plasmid* | This study | **K28091** |
| *S. cerevisiae*  *MAT alpha*  *Scc1(A547C)-pk6::Kan*  *Smc3 (E570C,S1043C)::ADE2*  *Smc1(G22c,K639C)::NAT*  *mrc1::His3MX 2.3kb Trp1-ARS1-Cen4 plasmid* | This study | **K28092** |
| *S. cerevisiae*  *MAT alpha*  *Scc1(A547C)-pk6::Kan*  *Smc3 (E570C,S1043C)::ADE2*  *Smc1(G22c,K639C)::NAT*  *csm3::His3MX 2.3kb Trp1-ARS1-Cen4 plasmid* | This study | **K28108** |
| *S. cerevisiae*  *MAT alpha*  *Scc1(A547C)-pk6::Kan*  *Smc3 (E570C,S1043C)::ADE2*  *Smc1(G22c,K639C)::NAT*  *ctf18::His3MX 2.3kb Trp1-ARS1-Cen4 plasmid* | This study | **K28115** |
| *S. cerevisiae MAT a*  *Smc1(G22C,K639C)::NatMX4*  *Smc3(E570C,S1043C)::ADE2*  *leu2::Gal-Scc1(R180E,R268D, A547C)-PK6::LEU2*  *chl1::KanMX*  *SCC3 (K404E)::HA3::HIS*  *TRP1-ARS1-CEN4 2.3KB plasmid* | This study | **K28175** |
| *S. cerevisiae MAT A*  *Smc1(G22C,K639C)::NatMX4*  *Smc3(E570C,S1043C)::ADE2*  *leu2::Gal-Scc1(R180E,R268D, A547C)-PK6::LEU2*  *SCC3 (K404E)::HA3::HIS*  *ctf4::KanMX*  *TRP1-ARS1-CEN4 2.3KB plasmid* | This study | **K28275** |
| *S. cerevisiae MAT A*  *Smc1(G22C,K639C)::NatMX4*  *Smc3(E570C,S1043C)::ADE2*  *leu2::Gal-Scc1(R180E,R268D, A547C)-PK6::LEU2*  *SCC3 (K404E)::HA3::HIS*  *mrc1::KanMX*  *TRP1-ARS1-CEN4 2.3KB plasmid* | This study | **K28278** |
| *S. cerevisiae MAT A*  *Smc1(G22C,K639C)::NatMX4*  *Smc3(E570C,S1043C)::ADE2*  *leu2::Gal-Scc1(R180E,R268D, A547C)-PK6::LEU2*  *SCC3 (K404E)::HA3::HIS*  *tof1::KanMX*  *TRP1-ARS1-CEN4 2.3KB plasmid* | This study | **K28280** |
| *S. cerevisiae MAT A*  *Smc1(G22C,K639C)::NatMX4*  *Smc3(E570C,S1043C)::ADE2*  *leu2::Gal-Scc1(R180E,R268D, A547C)-PK6::LEU2*  *SCC3 (K404E)::HA3::HIS*  *csm3::KanMX*  *TRP1-ARS1-CEN4 2.3KB plasmid* | This study | **K28282** |
| *S. cerevisiae MAT A*  *Smc1(G22C,K639C)::NatMX4*  *Smc3(E570C,S1043C)::ADE2*  *leu2::Gal-Scc1(R180E,R268D, A547C)-PK6::LEU2*  *SCC3 (K404E)::HA3::HIS*  *ctf18::KanMX*  *TRP1-ARS1-CEN4 2.3KB plasmid* | This study | **K28285** |
| *S. cerevisiae MAT a*  *Smc1(G22C,K639C)::NatMX4*  *Smc3(E570C,S1043C)::ADE2*  *leu2::Gal-Scc1(R180E,R268D, A547C)-PK6::LEU2*  *chl1::KanMX*  *ctf8-17::NATMX*  *SCC3 (K404E)::HA3::HIS*  *TRP1-ARS1-CEN4 2.3KB plasmid* | This study | **K28295** |
